# Delayed Iatrogenic Bladder Rupture Diagnosed by POCUS in the Emergency Department

**DOI:** 10.24908/pocus.v8i1.16239

**Published:** 2023-04-26

**Authors:** Helen J Lu, Edward H Lee, Stephen Alerhand

**Affiliations:** 1 Department of Emergency Medicine, Rutgers New Jersey Medical School Newark, NJ USA; 2 Department of Emergency Medicine, Hackensack University Medical Center Hackensack, NJ USA

**Keywords:** bladder rupture, point-of-care ultrasound, POCUS

## Abstract

Bladder rupture is an uncommon injury that leads to significant morbidity and mortality. Though occurring mostly due to trauma, this life-threatening pathology may also occur spontaneously or after a procedure such as transurethral resection of bladder tumor (TURBT). Computed tomography (CT) cystography is the standard imaging modality for diagnosis. However, this test is unlikely to be ordered in a patient with undifferentiated abdominal pain unless there is specific suspicion for this diagnosis. In our emergency department, a 48 year-old male with history of bladder cancer and TURBT two weeks prior to arrival presented with severe abdominal pain and difficulty urinating for 3 days. Point of care ultrasound (POCUS) revealed an irregularly shaped bladder, likely site of bladder rupture, and large amount of abdominal free fluid with sediment. These findings prompted an expedited diagnostic CT scan with cystography. Emergent exploratory laparotomy ultimately confirmed a small bladder defect with 2.5 L of urinary ascites. The diagnosis of non-traumatic bladder rupture can be overlooked in patients presenting with a peritonitic abdominen. The typically ordered test for such patients is standard CT, which carries a high false-negative rate for bladder rupture. This case highlights the utility of POCUS in facilitating a rapid diagnosis.

## Introduction

Bladder rupture is an uncommon injury associated with life-threatening peritonitis. Gross hematuria is the most common symptom, with an incidence of 77-100% 1. According to a Societe Internationale d’Urologie (SIU) consensus statement, patients may also present with suprapubic pain, inability to void, abdominal distention, and absent bowel sounds 2. Urinary ascites within the peritoneum can lead to uremia, metabolic derrangements, and pseudo-acute kidney injury from resorption of urea and creatinine by the peritoneum 3. Sepsis may also develop from urinary tract infection and intra-abdominal fluid collection from the bladder leak 4. If not promptly identified, intraperitoneal bladder rupture leads to significant morbidity and mortality 5. 

Bladder rupture occurs most commonly from blunt (67-86%) and penetrating (14-33%) trauma 6. It is often overlooked without concomitant pelvic fracture due to the bladder’s protection by the bony pelvis. Nevertheless, spontaneous bladder rupture may also occur, though with far rarer incidence of 1:126,000, and 25-80% mortality rate across case reports 7, 8, 9, 10. Rupture can occur spontaneously from lower urinary tract obstruction, malignancy, pregnancy, bladder dysfunction, pelvic radiotherapy, enema, and prior bladder or gynecologic surgery 4, 11, 12, 13. Rupture has even occurred due to the diuretic effects and decreased bladder filling sensation associated with alcohol intoxication 14. 

Trans-urethral resection of bladder tumor (TURBT) is the procedure of choice for the initial diagnosis, risk stratification, and management of bladder cancer 15. Unfortunately, bladder perforation may occur in 0.9-5% of cases^16^ from inadvertent full-thickness wall resection, perforation by resectoscope, or overdistention with saline. Among 4,144 patients who underwent TURBT, 0.36% required surgical intervention to repair a large perforation 17. Mean time from TURBT to diagnosis was 6.1 days, but 21 days in the two patients who died 17. Other patients with missed bladder ruptures have been discharged after initial surgery, but then presented later to the emergency department (ED) 18. 

The American Urological Association (AUA) reports that computed tomography (CT) cystography (using retrograde contrast instillation through a urethral catheter) is the diagnostic imaging test of choice to evaluate for bladder injury 19, with 94.7% sensitivity and 100% specificity in blunt trauma 20. However, to order this test requires a high level of suspicion for the rare diagnosis of bladder rupture. Furthermore, this time-consuming test requires bringing a potentially unstable patient outside the patient care area. Additionally, patients with an undifferentiated acute abdomen typically undergo conventional CT imaging, but the sensitivity for bladder injury is only 60% using this modality 20. 

Here we present the first known case of an iatrogenic bladder rupture diagnosed by point of care ultrasound (POCUS), as well as the first known case where delayed diagnosis of any bladder rupture was made using POCUS. This rapid bedside diagnosis led to prescient ordering of CT cystography and expedited definitive surgical management.

## Case Report

A 48 year-old male with history of bladder squamous cell carcinoma was triaged to the ED with worsening abdominal pain and difficulty urinating for 3 days. Triage vitals signs were notable for an elevated blood pressure of 178/89 mmHg and tachycardia to 110 beats per minute. Otherwise, the patient was afebrile with a temperature of 99.1°F, breathing at 20 breaths per minute, with an oxygen saturation of 98% on room air. As the patient looked uncomfortable upon arrival to the patient care area, he was attended to immediately by the physicians, while the nurse obtained intravenous access and blood tests. A brief history revealed that he had undergone TURBT six weeks earlier for initial evaluation and treatment of his bladder cancer, then another repeat TURBT two weeks prior to arrival for restaging and removal of the remaining tumor.

On physical examination, the patient was ill-appearing, tachycardic, and in significant distress due to abdominal pain. His abdomen was diffusely distended, tense, and tender to palpation with involuntary guarding. The rest of the physical examination (including genitourinary) was unremarkable.

**Figure 1:**
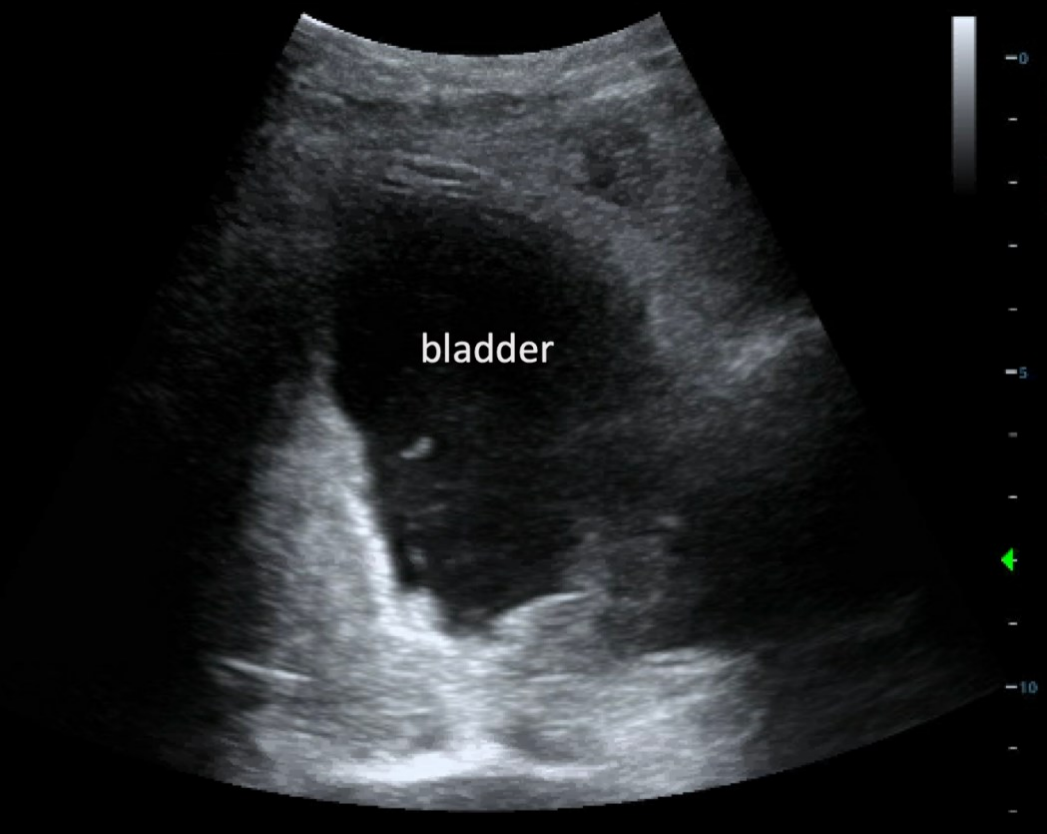
Point of care ultrasound in transverse plane demonstrating an irregularly-shaped bladder filled with anechoic contents and floating sediment.

**Figure 2:**
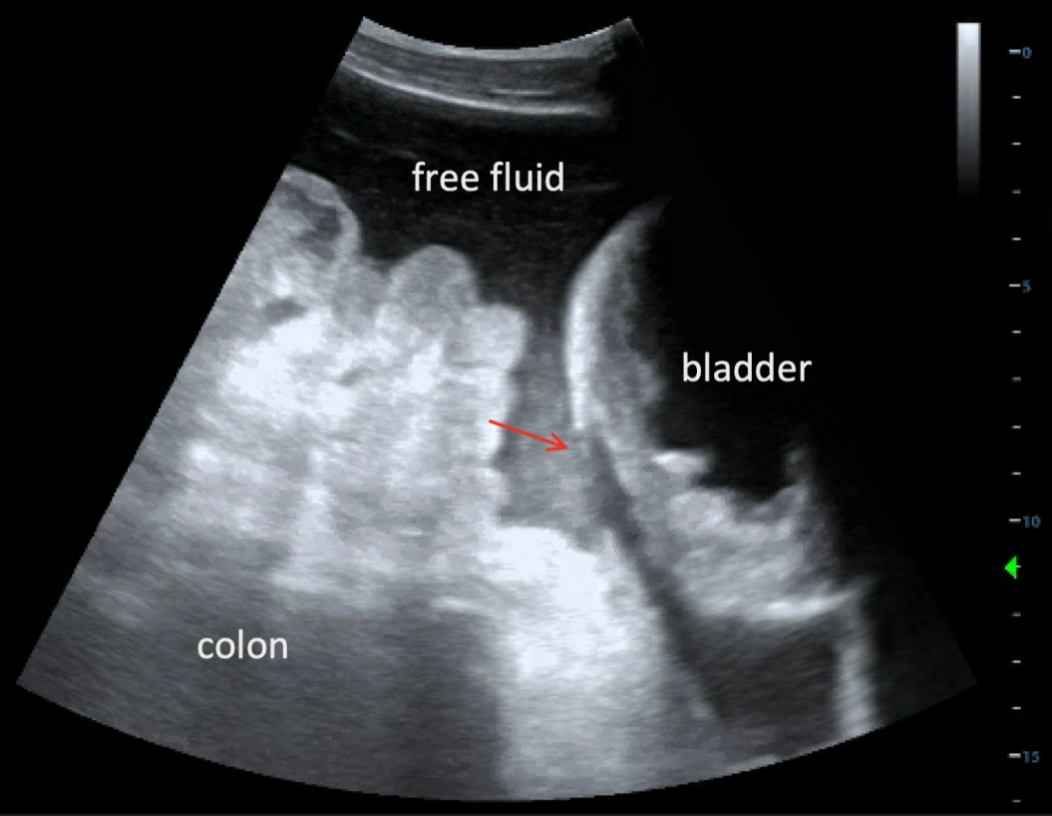
Point of care ultrasound in sagittal plane demonstrating the purported disruption in the hyperechoic bladder dome (red arrow).

**Figure 3:**
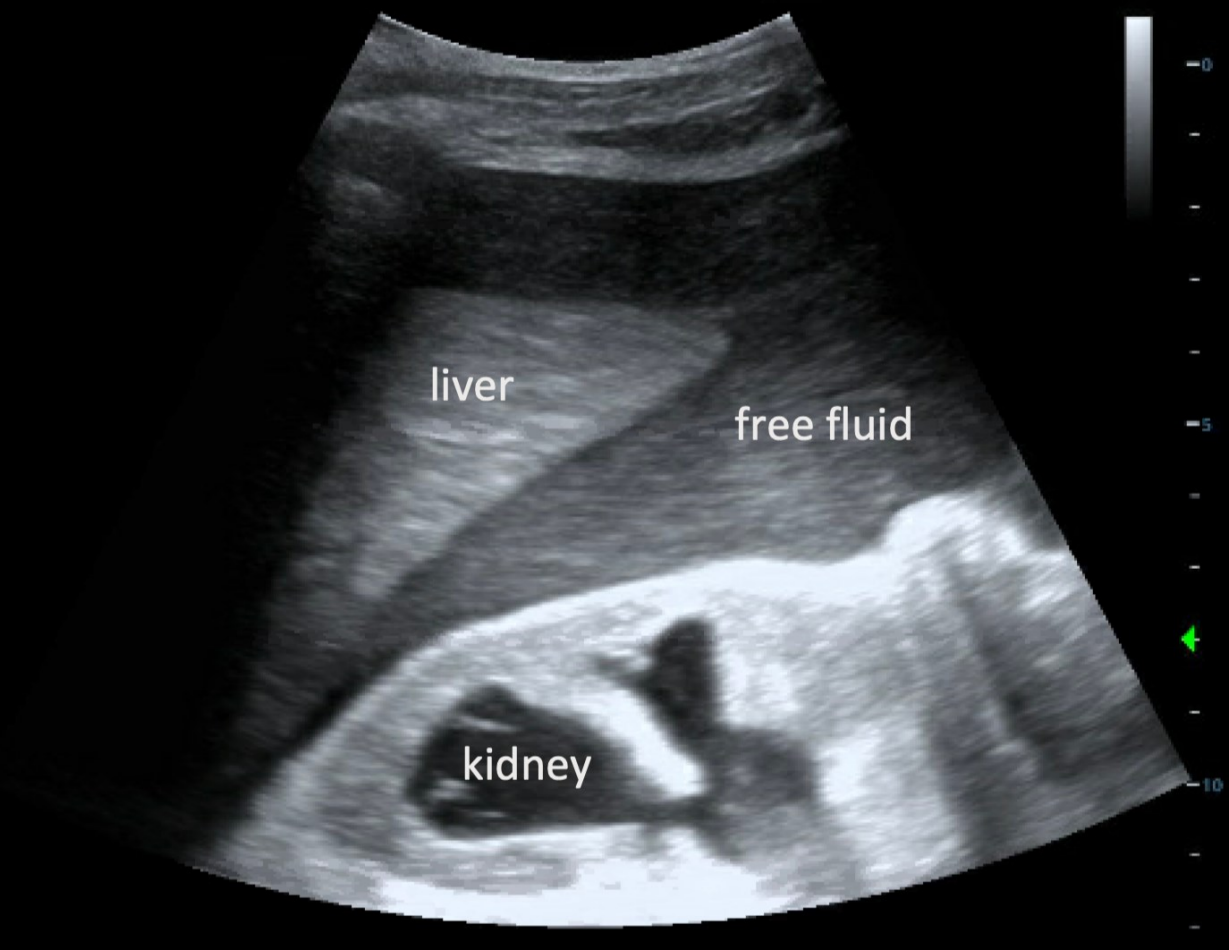
Point of care ultrasound in sagittal plane demonstrating large amount of free fluid in Morison’s pouch with moderate right-sided hydronephrosis.

An electrocardiogram from triage showed beginnings of a sine wave pattern, prolonged QRS interval to 160 msec, and peaked T waves altogether suggestive of hyperkalemia, for which appropriate medications were ordered. Given the patient’s urinary retention and history of prostate cancer, immediate foley catheter insertion was planned in order to drain an expected markedly distended bladder. However, POCUS performed at the bedside in the supine position revealed an irregularly-shaped, non-distended bladder containing anechoic fluid and floating sediment (Figure 1, Video S1). Disruption of the hyperechoic bladder dome was seen superiorly leading directly to intra-abdominal free fluid, indicating a likely site of bladder rupture (Figure 2, Video S2). The patient was then tilted into the Trendelenburg position to increase POCUS sensitivity for gravitationally-dependent free fluid. This evaluation revealed large volume of heterogenous free fluid throughout the right upper quadrant, as well as moderate bilateral hydronephrosis (Figure 3, Video S3). Altogether, this raised concern for intraperitoneal bladder rupture. 

**Figure 4:**
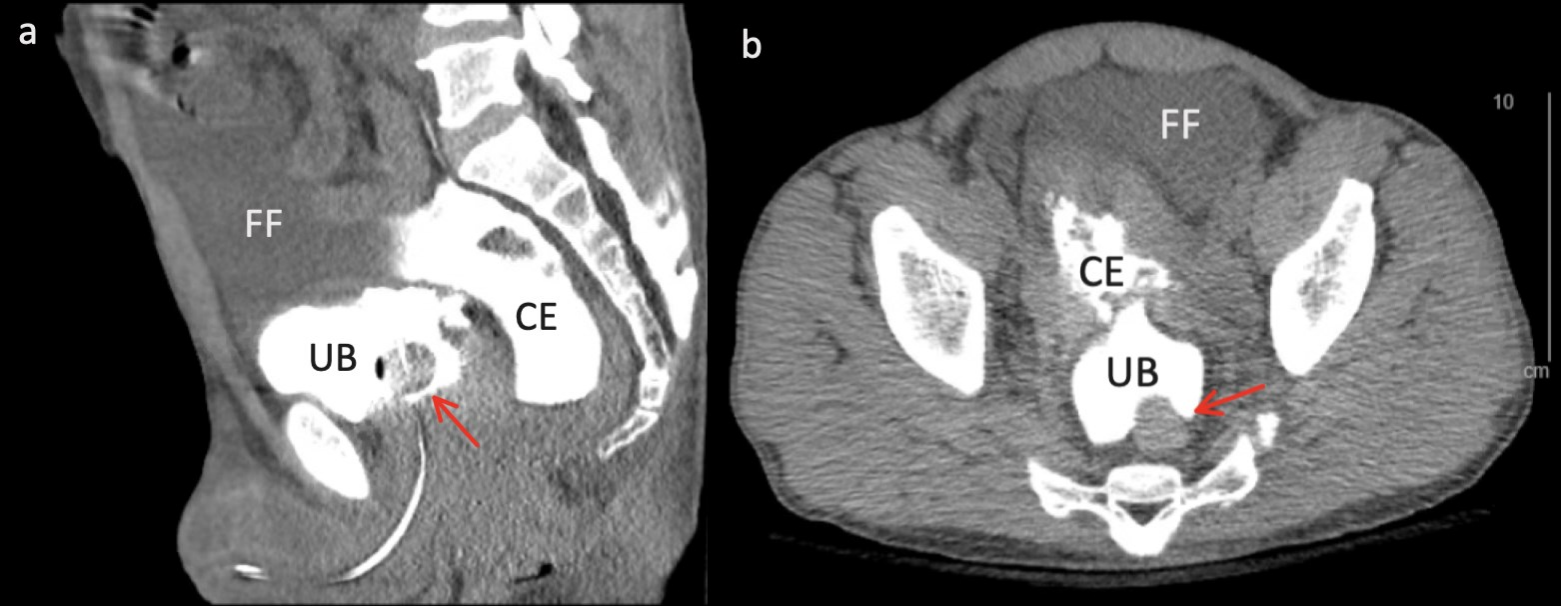
Computed tomography cystography demonstrating urinary contrast extravasation. In sagittal view (a), the Foley catheter appears filled with contrast. In both sagittal and axial (b) views, the catheter balloon (indicated by the red arrow) can be visualized within the urinary bladder (UB). Contrast extravasation (CE) can be seen emerging through the defect at the bladder dome and residing posteriorly in the supine patient. Eventually, the large amount of extravasated urinary contents has pushed the initial contents and free fluid (FF) into the anterior space as well.

The Urology service was consulted immediately and reviewed the POCUS images. Placement of a foley catheter drained cloudy yellow urine, and both services expedited CT scan of the abdomen and pelvis with cystography (Figure 4, Video S4). The suspected diagnosis was confirmed, and the patient was emergently taken directly from CT to operating room prior to any laboratory tests having resulted. During exploratory laparotomy, 2.5 liters of urinary ascites were evacuated from the peritoneal cavity, and the bladder wall defect was sutured closed. By this time, the initially drawn blood samples resulted with blood urea nitrogen of 180 mg/dL, creatinine of 12.4 mg/dL, potassium of 7.5 mEq/L, and anion-gap metabolic acidosis with pH of 7.23. Postoperative course was uneventful, acute kidney injury improved, and the patient followed up in clinic five days later for pelvic drain removal.

## Discussion

This is the first known case report in which an iatrogenic bladder rupture, and also a delayed rupture from any etiology, was diagnosed at bedside using POCUS. Based on POCUS findings, the clinicians specifically ordered CT cystography due to its higher sensitivity for bladder injury, instead of standard CT with intravenous contrast. This decision expedited definitive diagnosis and subsequent exploratory laparotomy by the Urology service. ED management saved the patient from significant risk of morbidity and mortality. In the large retrospective study of patients who underwent TURBT, two of the fifteen patients with bladder perforation who died from septic shock and multiorgan failure had prolonged time of 21 days from TURBT to diagnosis 17. 

Most prior cases of bladder rupture diagnosed by ultrasound occurred in the setting of trauma using the Focused Assessment with Sonography for Trauma (FAST) examination 21, 22, 23. A 2019 systematic review and meta-analysis reported that POCUS was 74% sensitive and 98% specific for intra-abdominal free fluid in trauma 24. Interestingly, these values did not differ greatly between hypotensive and normotensive patients. Abdominal free fluid was also reported in all traumatic and atraumatic case reports of intraperitoneal bladder rupture evaluated with radiologist- or technician-performed ultrasound 14, 21, 22, 23, 25, 26, 27. Similarly, our case showed large volume of free fluid in Morison’s pouch and the rectovesical pouch with the patient reclined in Trendelenburg position, which increases sensitivity for free fluid 28. 

The contour of a distended bladder would appear using 2D ultrasound as a roughly anechoic ellipsoid (not a circle), which explains why estimated bladder volume obtained from three dimensions is often multiplied by a correction coefficient factor of 0.72 29. Unexpectedly, our patient’s bladder appeared non-distended and irregularly-shaped, while containing anechoic fluid and floating sediment. In two other case reports of traumatic bladder rupture, the bladder also appeared irregularly-shaped using POCUS, with intraperitoneal free fluid as well 22, 25. 

Intraperitoneal bladder ruptures most commonly occur at the bladder dome, the weakest and most mobile part of the bladder 2. In one blunt trauma case, POCUS was used to identify the precise site of rupture at the bladder dome, leading directly to peritoneal free fluid 23. In our case, POCUS similarly identified a purported site of bladder rupture. Additionally, the Foley catheter balloon has also been shown to approximate anatomy using POCUS. In one atraumatic case of bladder rupture, the catheter was visualized using POCUS even though the bladder walls were not 27. In another bladder rupture case occurring after a fall, POCUS revealed that saline flushed through a catheter (with its bulb residing within the bladder) continued through a bladder dome defect directly into the peritoneum 21. 

## Why Should an Emergency Physician be Aware of This?

A bladder rupture occurs rarely and even less often from a non-traumatic etiology. A conventional CT scan ordered for a patient with an undifferentiated acute abdomen carries a high false-negative rate for this diagnosis. To order the gold standard CT cystography in the ED thus requires having a high level of suspicion. This case report highlights the utility of POCUS for rapidly identifying a bladder injury at the bedside, thereby leading to expedited appropriate follow-up imaging and transition to definitive surgical evacuation of free fluid and bladder wall repair.

Informed consent was obtained for use of the associated images and clips.

## Conflicts of Interest

HL, EL, and SA have no conflicts of interest to disclose.

## Supplementary Material 

Video S1 Point of care ultrasound in transverse plane demonstrating an irregularly-shaped bladder filled with anechoic contents and floating sediment.

Video S2 Point of care ultrasound in sagittal plane demonstrating the purported disruption in the hyperechoic bladder dome (red arrow).

Video S3 Point of care ultrasound in sagittal plane demonstrating large amount of free fluid in Morison’s pouch with moderate right-sided hydronephrosis.

Video S4 Computed tomography cystography demonstrating urinary contrast extravasation. In sagittal view, the Foley catheter appears filled with contrast. In both the sagittal and axial views, the catheter balloon can be visualized within the urinary bladder (UB). Contrast extravasation (CE) can be seen emerging through the defect at the bladder dome and residing posteriorly in the supine patient. Eventually, the large amount of extravasated urinary contents has pushed the initial contents and free fluid (FF) into the anterior space as well.
